# Postnatal Microstructural Developmental Trajectory of Corpus Callosum Subregions and Relationship to Clinical Factors in Very Preterm Infants

**DOI:** 10.1038/s41598-018-25245-7

**Published:** 2018-05-15

**Authors:** Radhika Teli, Margaret Hay, Alexa Hershey, Manoj Kumar, Han Yin, Nehal A. Parikh

**Affiliations:** 1 Perinatal Institute, Cincinnati Children’s Hospital Medical Center, Cincinnati, Ohio United States of America; 2 Department of Pediatrics, University of Cincinnati College of Medicine, Cincinnati, Ohio United States of America; 3 Center for Perinatal Research, Nationwide Children’s Hospital, Columbus, Ohio United States of America

## Abstract

Our objectives were to define the microstructural developmental trajectory of six corpus callosum subregions and identify perinatal clinical factors that influence early development of these subregions in very preterm infants. We performed a longitudinal cohort study of very preterm infants (32 weeks gestational age or younger) (N = 36) who underwent structural MRI and diffusion tensor imaging serially at four time points - before 32, 32, 38, and 52 weeks postmenstrual age. We divided the corpus callosum into six subregions, performed probabilistic tractography, and used linear mixed effects models to evaluate the influence of antecedent clinical factors on its microstructural growth trajectory. The genu and splenium demonstrated the most rapid developmental maturation, exhibited by a steep increase in fractional anisotropy. We identified several factors that favored greater corpus callosum microstructural development, including advancing postmenstrual age, higher birth weight, and college level or higher maternal education. Bronchopulmonary dysplasia, low 5-minute Apgar scores, caffeine therapy/apnea of prematurity and male sex were associated with reduced corpus callosum microstructural integrity/development over the first six months after very preterm birth. We identified a unique postnatal microstructural growth trajectory and associated clinical factor profile for each of the six corpus callosum subregions that is consistent with the heterogeneous functional role of these white matter subregions.

## Introduction

The corpus callosum (CC), consisting of millions of fibers, is the largest white matter structure of the brain. It is essential for integrating higher level (motor, sensory, and cognitive) functions between the cerebral hemispheres^1^. The CC is also one of the most adversely affected brain structures by premature birth, thereby contributing to a variety of neurodevelopmental impairments (NDI) that are frequently observed in very preterm infants^2,3^. Because CC fibers extend throughout different regions of the brain, its large structure can be subdivided into subregions that are structurally and functionally unique^1,4^. Most anatomists segment the CC into six to seven regions of interest (ROI) - splenium, isthmus, posterior midbody (PMB), anterior midbody (AMB), rostral body (RB), genu, and rostrum. Although the basic CC structure is complete by 18–20 weeks’ gestation, there is rapid growth during the third trimester, as well as up to two years postnatally. Weeks 23 to 33 of gestation form an especially critical window of development when various processes in the cytoarchitectural formation of the brain are active^5^. During this time, neuronal migration, formation of structural and functional connections, as well as active myelination are occurring throughout the brain^5^. Very preterm birth before 33 weeks’ gestation abruptly interrupts these processes, resulting in a considerably diminished rate of CC growth postnatally as compared to in utero. Aberrant microstructural development and/or direct injury to the CC results in weaker interhemispheric connections, which likely translates to poorer motor and cognitive function and neurodevelopmental disorders, such as cerebral palsy, later in life^2,3,6–13^. With the increasing prevalence of subtle brain injuries^8,14,15^ and continued high risk of NDI in very preterm infants, utilization of advanced imaging techniques, such as diffusion tensor imaging (DTI), is needed to understand the pathogenesis and facilitate sensitive detection of white matter abnormalities^16–19^.

Diffusion tensor imaging allows brain development to be studied *in vivo* based on the orientation and degree of water molecule diffusion that occurs through tissue fibers^20^. Well-established measures such as fractional anisotropy (FA) and mean diffusivity (MD) can be used as proxies of microstructural integrity of white matter fibers and serve as valuable diagnostic and prognostic biomarkers for detection of white matter injury/delayed brain maturation and prediction of NDI^18,21,22^. There are a limited number of studies that have used advanced MRI to examine the sub-segmental growth and development of the CC antenatally or soon after preterm birth^3,12,22–28^. Moreover, there are no studies that have examined sub-segmental CC microstructural development between 40 and 52 weeks postmenstrual age (PMA) and its relationship with perinatal risk factors in preterm infants during these first few months after birth. Assessment of CC microstructure is important in order to characterize its trajectory of myelination and axonal development during the most vulnerable period of brain development for preterm infants, as well as identify the impact of clinically modifiable extra-uterine factors^22,29,30^. Serial DTI studies that have examined the growth rate of the CC within the first few weeks after birth have also reported a positive association between reduced growth rate and delayed psychomotor development evaluated at 1 or 2 years of age^8,27^.

Previous studies have examined factors such as sex, birth weight, and degree of prematurity, and their effects on the growth and development of the CC either as a whole structure or at a single time point^3,4,23,25,31–36^. While other clinical antecedents, including bronchopulmonary dysplasia (BPD)^34,37–39^, caffeine therapy^33,38,40^ and breast milk, have been identified as factors that affect white matter development as a whole, the effect of these variables specifically on the early developmental trajectory of each CC subregion has not been studied in depth using advanced MRI techniques. To further our understanding of the clinical factors that influence the early longitudinal growth of each functionally distinct CC subregion, we performed up to 4 serial DTI exams in very preterm infants immediately after birth and extending up to six months after birth. Our objective was to perform probabilistic tractography and determine the early developmental trajectory of six CC subregions and the associated clinical factors that are protective or predispose this important white matter structure to injury or abnormal development in very preterm infants.

## Results

Of the 40 enrolled subjects, we excluded 4 because of poor image quality (subject motion artifact, etc.) or severe white matter injury (N = 2), parental request to withdraw infant from the study (N = 1) after the first MRI, or early death of the infant (N = 1) after the first MRI. For the 36 study infants, the mean (SD) gestational age was 26.9 (2.3) weeks, and birth weight was 983 (326) grams. Maternal demographics, as well as demographic and clinical characteristics of the infants, are summarized in Table 1. We performed 122 MRI scans; 20 at the first-time point, 36 at the second, 34 at the third, and 32 scans at the last time point. Twenty-two infants were scanned and had complete data at the 32, 38 and 52 weeks PMA time points.

**Table 1:** Demographic and clinical characteristics of participating subjects and their mothers.

Clinical Factor	Mean (SD) or N (%)
Maternal age*	26.0 (6.3)
Maternal education, college degree or greater*	15 (42.9%)
Gestational age, weeks	26.9 (2.3)
Birth weight, grams	983 (326)
Low Apgar score at 5 min (≤5)*	15 (42.9%)
Male sex	15 (41.7%)
Bronchopulmonary dysplasia	27 (75.0%)
Total days of breast milk received in the first 28 days after birth	22 (6.7)
Caffeine therapy duration, days	52 (20.5)
PMA at MRI scan #1, weeks	28.9 (1.1)
PMA at MRI scan #2, weeks	32.6 (1.0)
PMA at MRI scan #3, weeks	39.4 (1.3)
PMA at MRI scan #4, weeks	52.7 (1.5)

*Data unavailable for one infant due to home birth.

Of the 36 infants in the final cohort, brain injury or delayed development, as evaluated using our standardized previously published MRI scoring system^41^, was not present in 11, mild in 19, and moderate in 6 on one or more structural brain MRI exams up to 52 weeks PMA. None of the infants exhibited injury to the CC. Of the 6 cases read as having moderate degree of injury/delayed development, 4 infants had sequelae of prior intraventricular hemorrhage with secondary ventriculomegaly and mild to moderate degree of brain volume loss/delay. One infant had more than a four weeks delay in gray and white matter maturation with brain volume loss/delay and one infant exhibited diffuse excessive high signal intensity in multiple white matter regions with mild brain volume loss/delay.

### Inter-rater Reliability

The ICC values for the PMB and isthmus ranged from 0.87 to 0.95 for the FA and MD measurements. Because evaluating ICC alone can be misleading, Table 2 also reports more robust measures such as the within-subject SD and repeatability^42^.

**Table 2:** Inter-rater reliability measures for the posterior midbody and isthmus subregions of the corpus callosum.

	PMB FA	PMB MD	Isthmus FA	Isthmus MD
Absolute agreement ICC (95% CI)	0.94 (0.82, 0.98)	0.93 (0.81, 0.98)	0.92 (0.75, 0.97)	0.95 (0.86, 0.98)
Consistency agreement ICC (95% CI)	0.88 (0.68, 0.96)	0.87 (0.66, 0.96)	0.87 (0.67, 0.96)	0.92 (0.78, 0.97)
Within Subject SD	0.0079	3.87 × 10^−05^	0.0106	5.13 × 10^−05^
Repeatability	0.0220	1.07 × 10^−04^	0.0293	1.42 × 10^−04^

ICC – Intraclass correlation coefficient; SD – standard deviation; PMB – posterior midbody; FA – fractional anisotropy; MD – mean diffusivity.

### CC Microstructural Growth Trajectory

**Figure 1:**
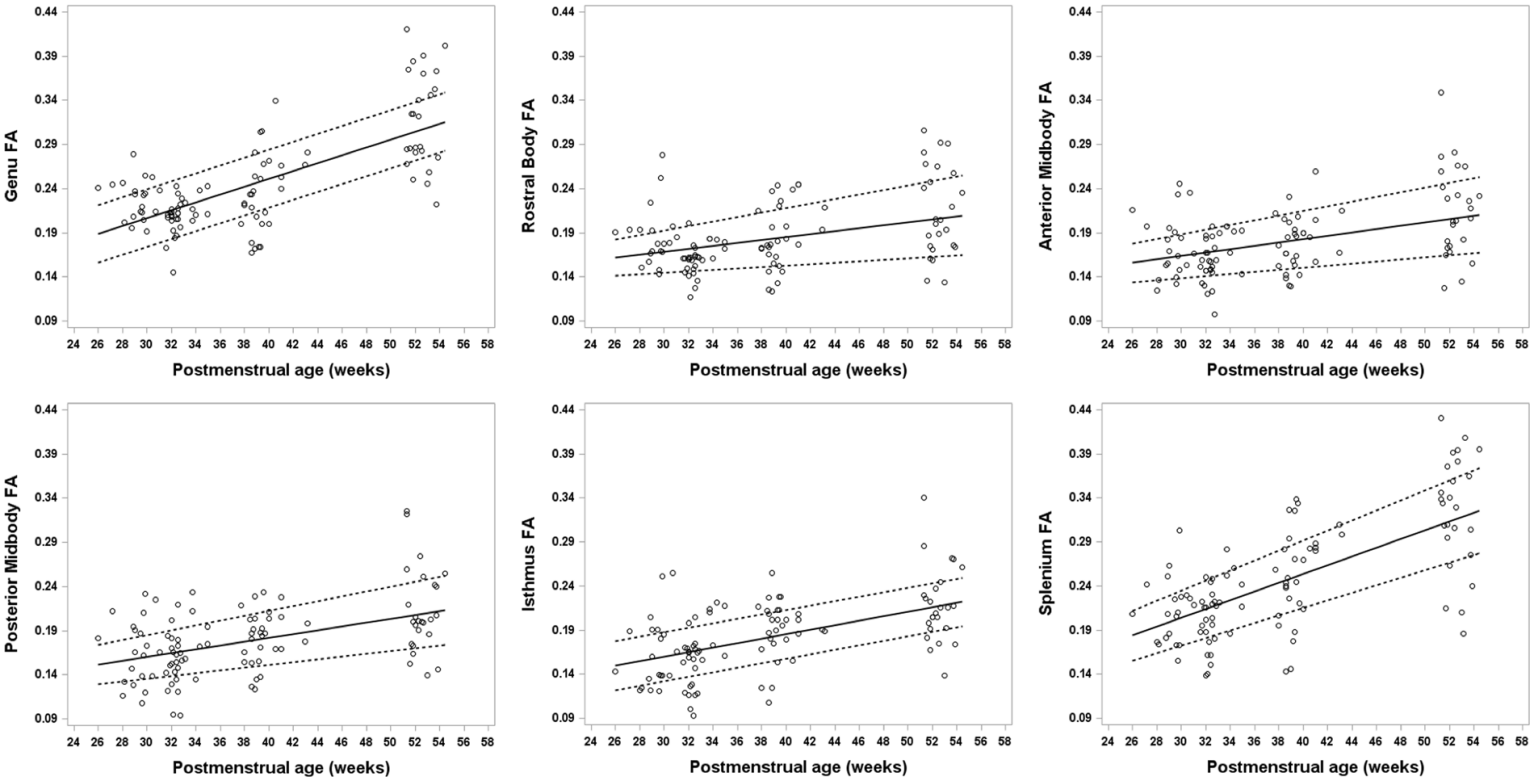
Developmental trajectory of fractional anisotropy (FA) changes for all six subregions of the CC ranging from about 26 weeks to 54 weeks postmenstrual age (PMA) in very preterm infants. A general increase in FA is evident in each CC subregion with advancing PMA during the first few months after birth. A greater increase over time is noted in the splenium and genu.

**Figure 2:**
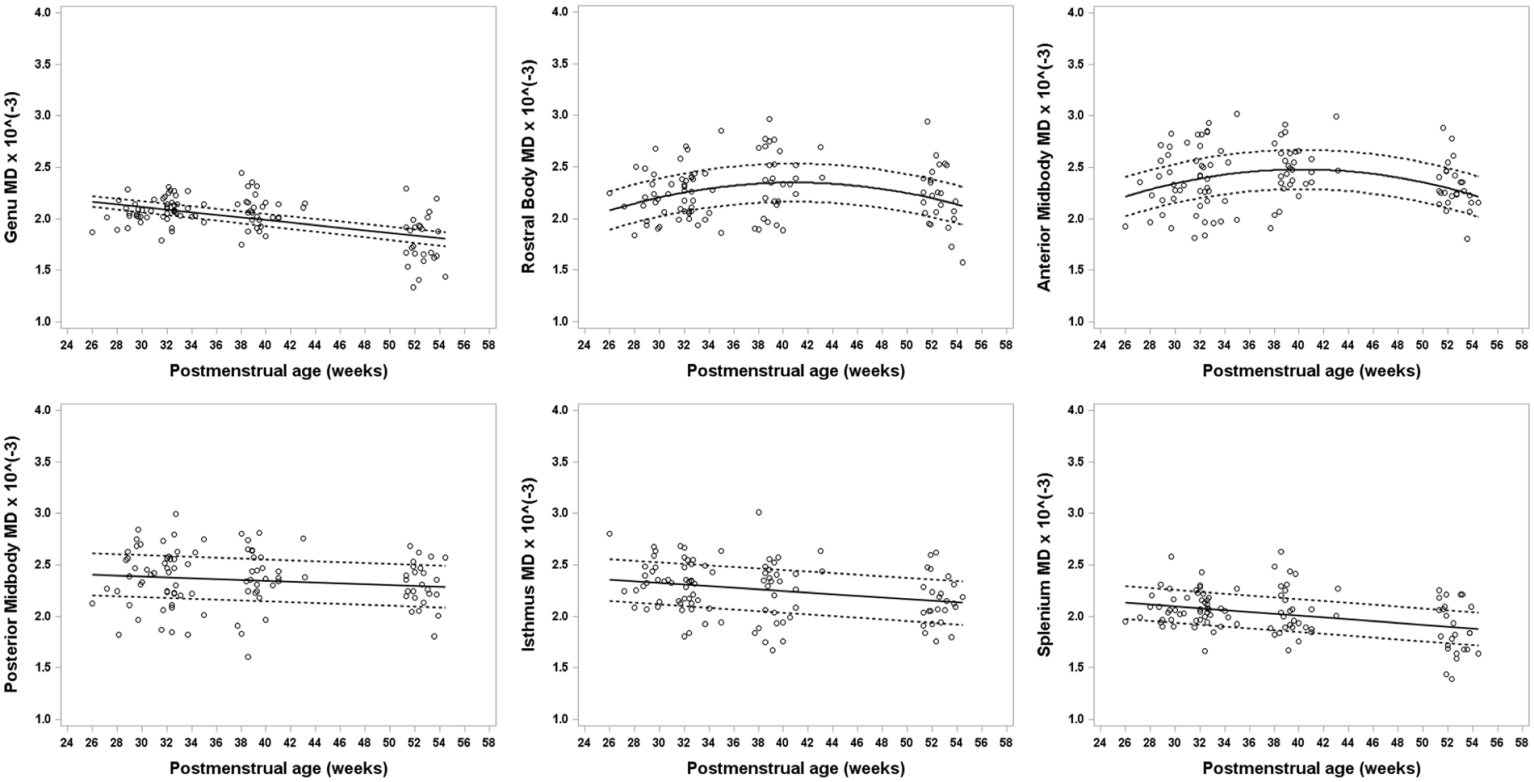
Developmental trajectory of mean diffusivity (MD) changes for all six subregions of the CC ranging from about 26 weeks to 54 weeks postmenstrual age (PMA) in very preterm infants. An overall decrease in MD values is evident in the different subregions of the CC, except anterior midbody and rostral body, as PMA increases in the first few months after birth. For the anterior midbody and rostral body, MD initially increases until about 40 weeks PMA, after which MD decreases. MD, also known as the apparent diffusion coefficient (ADC) is an average of the three eigenvalues (λ_1_, λ_2_, λ_3_).

**Figure 3:**
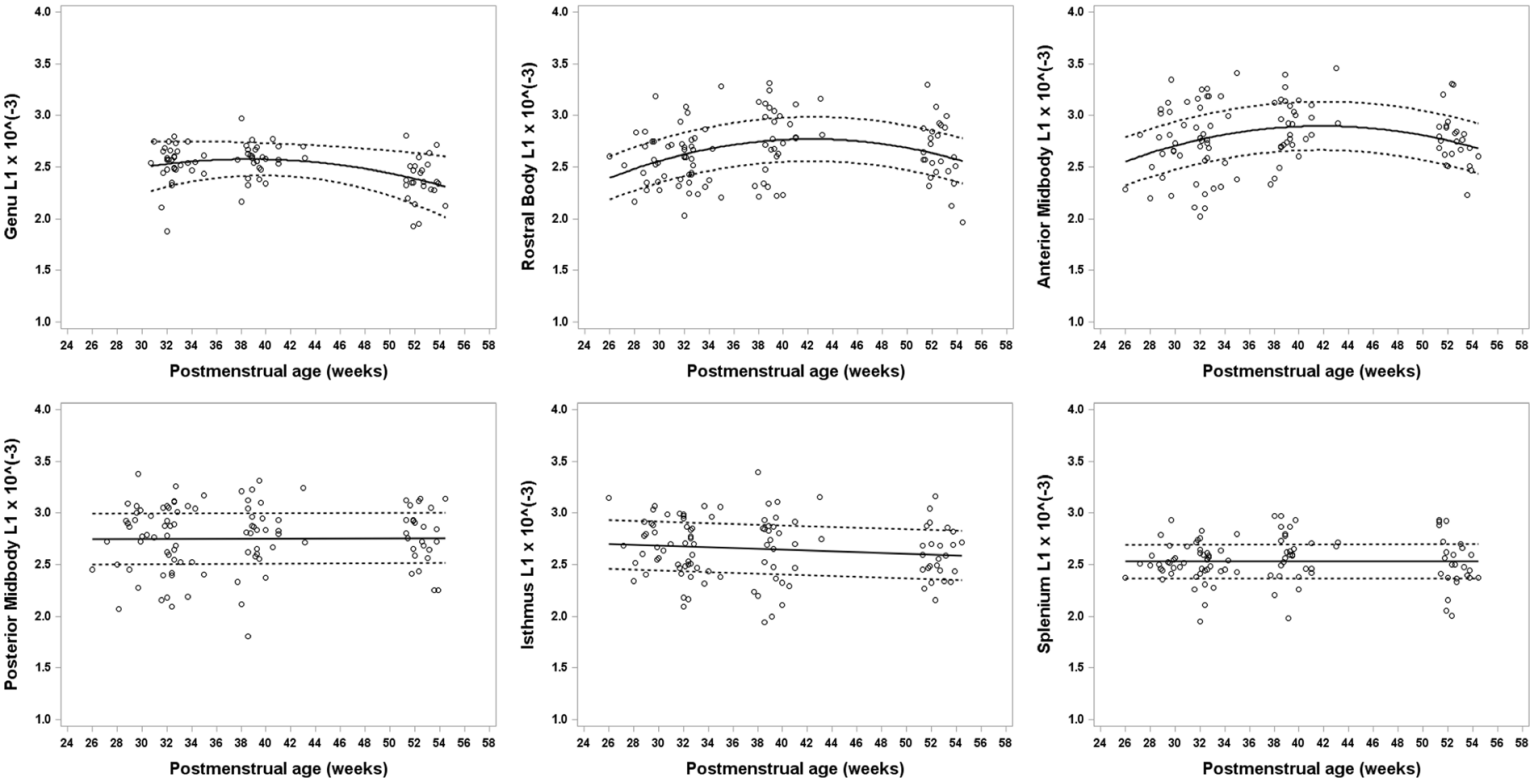
Developmental trajectory of axial diffusivity (AD; L1) changes for all six subregions of the CC ranging from about 26 weeks to 54 weeks postmenstrual age (PMA) in very preterm infants. Axial diffusivity values, also known as λ_1_, remained stable over the first few months in the splenium, isthmus, and posterior midbody. Conversely for segments of the anterior half of the CC, AD remained stable or increased initially until around 40 weeks PMA, after which AD decreases.

**Figure 4:**
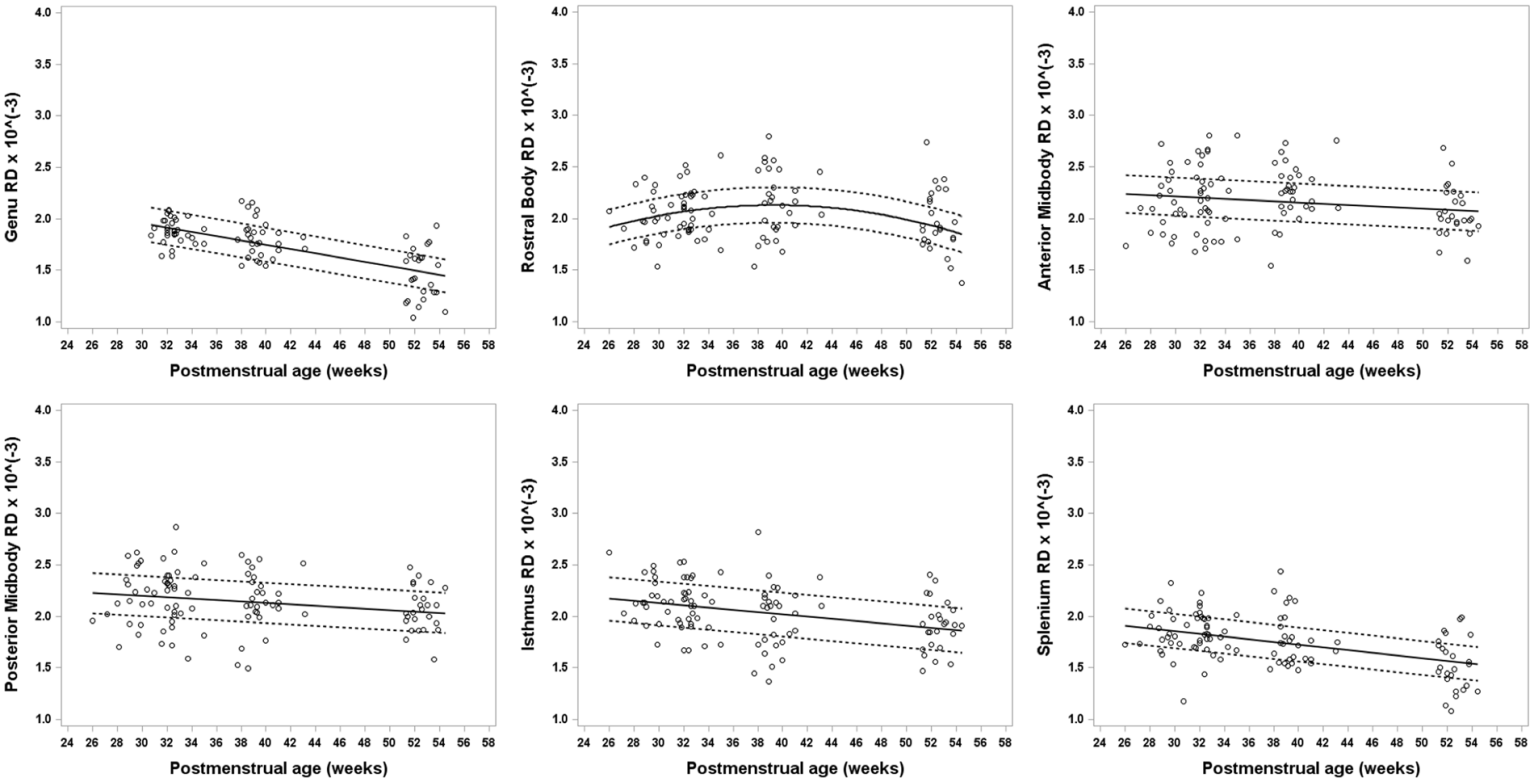
Developmental trajectory of radial diffusivity (RD) changes for all six subregions of the CC ranging from about 26 weeks to 54 weeks postmenstrual age (PMA) in very preterm infants. Radial diffusivity values, the average of λ_2_ and λ_3_, significantly decrease in the splenium, isthmus, anterior midbody (AMB), posterior midbody (PMB), rostral body (RB), and especially in the genu with advancing PMA. RD in the RB increased initially until around 40 weeks PMA, after which RD decreases. The trajectories for RD values for the six segments of the CC over the first 6 months of development appear very similar to MD.

The microstructural growth trajectory of the CC subregions differed considerably based on the four DTI scalars. In general, FA increased while MD, AD, and RD decreased or remained stable over the first six months after birth (Figs 1–4). The trajectory of brain maturation, as defined by FA, increased between 26 weeks and 54 weeks PMA and was considerably greater for the genu and splenium as compared to the RB, AMB, PMB, and isthmus. These latter four CC subregions displayed a very similar FA trajectory (Fig. 1), and each was also significantly influenced by PMA (p < 0.001). Postmenstrual age also exhibited a significant relationship with MD of each of the six subregions (p < 0.001). Mean diffusivity of the RB and AMB displayed an upward trajectory until 40 weeks, followed by a decrease, while the remaining four CC subregions showed a decline over the first 6 months after birth (Fig. 2). Axial diffusivity increased until 40 weeks followed by a decrease for the anterior three subregions of the CC, while the posterior three showed little change over time and no correlation with PMA (Fig. 3). The trajectory of RD values closely resembled the trajectory of MD values for each CC subregion (Fig. 4).

### Clinical Antecedents

We identified multiple clinical antecedents of the four DTI scalars for each subregion of the CC. Increasing FA was significantly and directly correlated with increasing PMA and higher birth weight for each of the six CC subregions (p < 0.05 to p < 0.001); Fig. 1; Table 3). Birth weight was also significantly but inversely correlated with RD of the genu and splenium ((p < 0.05; Table 3). A small 100-gram increase in birth weight was equivalent to two weeks of microstructural maturation of the splenium as measured by RD. Decreased microstructural development of the splenium was also associated with a low Apgar score at 5 minutes and maternal age, with advancing age inversely associated with FA. For the genu, only PMA and birth weight were directly associated with FA and inversely associated with RD; PMA was positively associated with AD. Maternal education (college level or higher) and survival without BPD exhibited the largest correlation (negative) with radial and axial diffusivity measures of microstructural development. Sub-college level education was adversely correlated with both RD and AD of the AMB and RB, while survival without BPD was associated with greater maturation of the RB, as measured by both RD and AD. Total days of caffeine therapy was positively associated with AD and RD of the AMB subregion. Male sex was directly and significantly associated with RD and AD, suggesting poorer/slower maturation of the PMB and AMB subregions (p < 0.05). We did not find significant associations between duration of breast milk received in the first 28 days and microstructural measures of any of the CC subregions. We found nearly the same risk factors correlated with MD of a given CC subregion as correlated with AD and RD for that subregion (Table 3). This is not surprising given that MD is mathematically related to AD and RD.

**Table 3:** Clinical factors as they independently relate to FA, RD, and AD values of each corpus callosum subregion.

CC Subregion	Mean FA Difference (95% CI)	Mean RD Difference (95% CI)	Mean AD Difference (95% CI)
Splenium	PMA 0.00472 (0.00371, 0.00569)*** MA −0.00172 (−0.00313, −0.000318)* BW 0.00609 (0.00303, 0.00918)*	PMA −0.0121 (−0.0162, −0.00810)*** BW −0.023 (−0.0393, −0.00673)* Apgar 0.167 (0.0654, 0.268)*	Apgar 0.142 (0.0491, 0.235)*
Isthmus	PMA 0.00237 (0.00176, 0.00298)*** BW 0.00470 (0.00182, 0.0076)*	PMA −0.01057 (−0.0156, −0.0055)**	
PMB	PMA 0.002149 (0.00135, 0.00295)*** BW 0.00374 (0.000937, 0.0065)*	PMA −0.00672 (−0.0109, −0.00253)* Male 0.182 (0.0449, 0.319)*	Male 0.188 (0.0219, 0.354)*
AMB	PMA 0.00183 (0.000914, 0.00274)* BW 0.00333 (0.0059, 0.0308)*	PMA 0.02608 (0.00827, 0.0439)* PMA^2^ −0.00122 (−0.00191, −0.00053)* DCT 0.00339 (0.000954, 0.0058)* EDU 0.1353 (0.0258, 0.2448)* Apgar 0.2275 (0.1249, 0.33)** Male 0.1387 (0.0268, 0.2506*	PMA 0.0378 (0.0149, 0.0607)* PMA^2^ −0.00142 (−0.00231, −0.00053)* DCT 0.00335 (0.000197, 0.0065)* EDU 0.180 (0.0505, 0.310)* Apgar 0.165 (0.0669)*
RB	PMA 0.00157 (0.000611, 0.00254)* BW 0.00342 (0.0011, 0.00575)*	PMA 0.0308 (0.0154, 0.0462)** PMA^2^ −0.00138 (−0.00198, −0.00078)***EDU 0.1343 (0.0159, 0.253)* No BPD −0.2227 (−0.364, −0.0812)* Apgar 0.167 (0.0493, 0.285)*	PMA 0.04211 (0.0341, 0.296)*** PMA^2^ −0.00155 (−0.00230, −0.00080)** EDU 0.1547 (0.0158, 0.294)* No BPD −0.228 (−0.395, −0.0608)*
Genu	PMA 0.004377 (0.00355, 0.0052)*** BW 0.00350 (0.000638, 0.00636)*	PMA −0.02072 (−0.0251, −0.0163)*** BW −0.015 (−0.0287, −0.00128)*	PMA 0.02403 (0.00398, 0.0441)* PMA^2^ −0.00119 (−0.00189, −0.00049)*

*P < 0.05, **P < 0.01, ***P < 0.001

Note: All RD and AD values should be multiplied by 10^−3^. Coefficient for BW represents a difference in birth weight of 100 grams. Abbreviations: CC: corpus callosum; CI: confidence interval; PMB: posterior midbody of CC; AMB: anterior midbody of CC; RB: rostral body of CC; PMA: postmenstrual age at MRI scan; PMA^2^ quadratic term of PMA; BW: birth weight; MA: maternal age; Apgar: score ≤5 at 5 minutes after birth; DCT: total number of days on caffeine therapy; EDU: maternal education level (below college level); BPD: bronchopulmonary dysplasia.

## Discussion

We employed serial DTI studies, commencing soon after birth, to examine the developmental trajectory of six structurally/functionally unique subregions of the CC over the first six months following very preterm birth. We used DTI parameters, including FA, MD, AD, and RD, to serve as important surrogate measures for white matter microstructural integrity. We performed serial DTI scans over the critical window of the third trimester, when very preterm babies are most vulnerable while receiving care in the neonatal intensive care unit, and over the first few months after discharge, when plasticity and recovery from earlier insults are feasible. Additionally, we identified several important antenatal and neonatal clinical factors that significantly impact CC bundle development, as evidenced by *in vivo* surrogate measures.

Yakovlev and Lecours as well as Gilles studied myelination in infancy using detailed histological methods of white matter staining^43,44^. These seminal studies identified that myelination of each brain structure varies in chronology and tempo. Yakovlev and Lecours’ work suggested that the commissural zones of the forebrain display the longest processes of myelination and begin to show evidence of myelination (identified with Weigart’s hematoxylin preparations) at 16 weeks postnatal^43^. Furthermore, Brody *et al*. reported myelination of the CC occurs most rapidly in the first 2 years^45^. Following a comprehensive review, Dubois *et al*. hypothesized that early white matter development progresses through three stages – fiber organization, “pre-myelination”, and “true” myelination – that are characterized by a unique set of changes in DTI parameters. It is now well-established that overall, FA measures the anisotropy of water within white matter bundles, while the diffusivity metrics indicate brain water content and membrane density^46^. However, it is likely that compaction and fiber diameter also contribute to these microstructural measures.

An increase in FA during the third stage indicates “true” axonal myelination and decreased membrane permeability^19,44–47^. This last stage occurs in the CC mid-body first at round 40 weeks PMA, followed by myelination of the splenium and finally the genu within the first few postnatal months, making it a particularly crucial time period for proper maturation of the largest white matter bundle^44^. This is consistent with Gilles’ overall summary that the first signs of corpus callosum (central part) myelination occurs during weeks 36 to 40 of gestation^44^. Additionally, the sequence of myelination identified by Yakovlev and Lecours also suggested that myelination of the corpus callosum begins with the splenium at 16 weeks postnatal and gradually progresses in the rostral direction. However, both Gilles’ and Yakovlev and Lecours’ groups suggest that the majority of corpus collosum myelination occurs in the early postnatal months, extending into the first decade of life^43–45^. Our data revealed a steep increase of FA values with time in the splenium and genu, which may suggest this third stage of white matter maturation. FA in all six subregions of the CC showed a statistically significant increase over time. Furthermore, the first stage involves more directionally organized axonal fibers, which can be expressed by increased anisotropy and decreased mean and radial diffusivity but is distinguished by an increase in axial/parallel diffusivity^19^. We observed greater change in RD than AD over time, consistent with prior evidence in other white matter tracks^19,48^. Overall, RD in all subregions of the CC showed a statistically significant decrease with time. This is consistent with the assumption of a cylindrically symmetric decrease in diffusion due to myelination^22,47,48^.

We only observed an increase in AD in the RB and AMB between approximately 28 and 40 weeks PMA, after which AD began to decrease. Axial diffusivity in the remaining four CC subregions remained stable or decreased slightly over time suggesting this transient increase in the mid-body of the CC likely reflected the presence of crossing fibers (e.g. projection fibers) rather than fiber organization^46,49^. This would also explain the transient rise in RD during the same time period and within the same CC subregions. One study also found that because crossing fibers can sometimes cause such fictitious changes in AD and RD based on the degree of pathology of the underlying tissue, white matter development is more holistically characterized by changes in FA and MD values^49^. The above changes would suggest that by 26 to 28 weeks gestational age, “pre-myelination” of the CC is already underway. During this second stage, there is a decrease in overall water diffusivity as glial cells and oligodendrocyte lineage precursors multiply^46^. Considering this chronology and its reflection in DTI measures allowed us to take a more objective look at the impact of prematurity on each specific region of the CC when measuring the developmental growth trajectory^22,24,50,51^.

A recent functional topography study utilized fMRI and DTI to map the functional/structural differences between these specific subregions of the CC in healthy adults and patients with callosotomy^1^. This study found that the anterior CC is active during taste stimuli, the central portion is responsible for motor tasks, and the central and posterior subregions play a role in tactile stimulation. More specifically, the splenium is responsible for integrating and communicating auditory and visual information. Using DTI tracking, the researchers were also able to show crossing fibers through the different CC subregions. The genu is also known to be involved in working memory, and this is associated with higher FA and lower RD values within the tract^22^. These studies extend what is already known about infants and children with injury or developmental abnormality of the CC. Functionally, these typically manifest as delays in walking, talking, or reading, and clumsiness and poor motor coordination, particularly on skills that require coordinated use of both hands or legs. Data analysis in our study revealed trends linking each clinical factor exclusively to a specific segment or group of segments within the corpus callosum, further supporting the notion that each subregion of the CC has both structural and functional heterogeneity^1,4^.

Between 26 and 54 weeks PMA, FA values for all six CC subregions, especially in the splenium and genu, increased as gestational age increased, suggesting rapidly increasing white matter microstructural integrity and maturation. Not surprisingly, this same relationship, albeit not as strong, was evident between birth weight and FA as well. A significant relationship was also found between PMA and AD for the AMB, RB, and genu subregions and RD for all six CC subregions. These relationships may have been influenced by crossing fibers through these subregions of the CC, thereby falsely lowering AD and RD values. Decreasing diffusivity values over time denotes growth in white matter cyto-architecture. Our results, overall, are consistent with previous studies that have identified a similar positive relationship between gestational age and white matter growth in terms of fractional anisotropy and diffusivity^2–4,12,26,38^. Our results also suggest that the genu and splenium are strongly influenced by age and birth weight, and the splenium is additionally associated with illness severity/need for resuscitation at birth, as reflected by low 5-minute Apgar scores. The process of white matter maturation, notably in the anterior- and posterior-most regions of the CC, is especially vulnerable to delay if the infant prematurely enters the extra-uterine environment^22,38^.

The relationship between maternal education level and white matter development has not been extensively studied. We found that a below-college degree maternal education level was significantly associated with increased diffusivity values of the AMB and RB, suggesting delays in maturation for these CC subregions. Delayed maturation of these CC subregions may in part explain the well-known association between maternal socioeconomic status, especially lower maternal education, and poorer cognitive and behavioral outcomes in preterm infants^52,53^. Larger studies examining whole brain connectivity are warranted to further elucidate the structural basis for poor academic outcomes of infants born to mothers with lower educational status.

We found a significant direct correlation between increasing exposure to caffeine therapy for apnea of prematurity and axial and radial diffusivity of the AMB. No significant correlation was found between the duration of breast milk and CC development (FA and diffusivity measures). Our group and others have examined the effects of caffeine therapy and breast milk on neonatal brain development^33,38,40,54^. These studies found that a longer duration of human milk, as well as caffeine therapy, were strongly associated with greater CC maturation^38,40^. This difference in findings might be explained by our smaller sample size or because all subjects in our cohort were administered both caffeine therapy and breast milk to some extent, and this lack of heterogeneity may have obscured the true relationship between these factors and CC maturation. Caffeine therapy may also be serving as a proxy for infants with more severe apnea of prematurity, a condition associated with worse neurodevelopment.

Infants that survived without BPD had lower diffusivity in the RB, signifying greater fiber integrity when compared to subjects diagnosed with BPD. This finding supported our hypothesis, which was based on previous studies that identified an association between chronic neonatal respiratory disease and white matter abnormalities^28,37,38,55,56^. One study examined whole brain white matter using an automated observer independent method called tract-based spatial statistics and found that preterm infants given mechanical ventilation for more than 2 days in the perinatal period exhibited decreased FA values in the genu of the CC^55^. Another group found that chronic lung disease and postnatal infection in preterm infants correlated with decreased FA in the CC as a whole, as well as the posterior limb of internal capsule^56^. Additionally, 15 very preterm infants in our cohort were born with an Apgar scores ≤5 at 5 minutes. These infants exhibited greater diffusivity values in the splenium, AMB, and RB, implying lesser overall CC development as compared to infants with higher 5 minute Apgar scores. While an association between low Apgar scores and microstructure in six brain regions (genu and splenium of CC, anterior and posterior limbs of the internal capsule, thalamus, and globus pallidus) has been reported^34^, no prior study has reported an association between low Apgar scores and CC subsegmental development during the first few months of life.

It is now well established that preterm females exhibit lower rates of NDI than their male counterparts, though the reasons for this are not yet well characterized^57,58^. When compared to female infants, males had greater axial and radial diffusivity in the PMB. Previously, Rose *et al*. identified delayed splenium development (lower FA and higher MD) on DTI at term-equivalent age in males as compared to females (PMB and other parts of the CC body were not examined)^10^. Fractional anisotropy in the splenium also correlated with abnormal neurodevelopment at 18 to 22 months corrected age. Other groups have also found that the growth of the CC in the first 2 weeks after birth was poorer among males compared to females^2^, and male sex was correlated with a smaller PMB^3^. These findings suggest that sex differences in the maturation of the posterior subregion of the CC may partially explain the vulnerability of very preterm boys to higher rates of NDI.

Our study had a few limitations. Although we performed between two to four MRI scans per infant and a total of over 100 MRI scans, we studied a relatively small number of infants. In addition to imaging infants using a higher resolution 3 T MRI scanner, we employed probabilistic tractography methods to improve our ability to generate accurate CC segmental tracts, which tend to reside in regions of multiple crossing fibers, and to quantify developmental changes in the white matter tracts^17,30,59^. Recent advances in acquisition of higher order diffusion model data as well as multi b-value/multi-shell diffusion MRI in neonates will facilitate more robust studies to sensitively assess brain cellular compartment models in the developing very preterm brain^60^.

Overall, we identified several factors that favored greater CC microstructural development, including advancing age, higher birth weight, and college level or higher maternal education. Bronchopulmonary dysplasia, low 5 minute Apgar scores, and male gender were associated with reduced CC microstructural integrity/development over the first six months after very preterm birth. Because the CC is involved with important psychomotor functions and hemispheric communication, damage or persistently delayed development is likely an important contributor to adverse motor, cognitive and behavioral long-term outcomes^61^. A more accurate mapping of the structural and functional division of the CC will lay the foundation for early prediction of developmental disabilities in populations at high risk for brain injury and delayed brain development. We are currently following this cohort to 2 years corrected age to measure these functional outcomes and determine if detailed tractography measures of CC subregions can serve as important prognostic biomarkers of NDI.

## Methods

### Subjects

In this longitudinal cohort study, 40 very preterm infants born at less than or equal to 32 weeks of gestation were enrolled. All very preterm infants from the Neonatal Intensive Care Unit at Nationwide Children’s Hospital, Columbus, Ohio from 8/2012 to 6/2014 were eligible for recruitment. We excluded subjects with any known congenital anomalies of the central nervous system or cyanotic heart disease. Additionally, infants on very high mechanical ventilator support (e.g. peak inspiratory pressure >30 and/or Fraction of inspired oxygen >50%) within the first 28 days after birth were considered ineligible for the study due to increased risk associated with moving them to MRI. We performed three advanced MRI scans (structural and functional imaging) at 32 weeks, 38 weeks, and 52 weeks PMA. We also performed a fourth MRI for infants <30 weeks gestational age, between 2 and 4 weeks after birth. For all inpatient scans – typically the first three MRI time points – we used a 3 Tesla-MRI compatible transport incubator (Nomag 3.0IC, Lammers Medical Technology, Germany) to minimize handling and enhance safety. Two neonatal research nurses and a neonatologist accompanied each study infant to MRI. One neonatal research nurse was present for outpatient scans. Before each MRI, infants were fed (if not NPO) and swaddled to induce natural sleep; sedation was not used under any circumstance. To protect the infants from MRI noise, the subjects’ ears were protected with Insta-Putty Silicone Ear Plugs (E.A.R. Inc, Boulder, CO) and Natus Mini Muffs (Natus Medical Inc, San Carlos, CA). We continuously monitored all infants during scanning for desaturations, bradycardias, or any movements. The same MR technologist/physicist conducted all imaging. We obtained approval for this study from the Institutional Review Board of Nationwide Children’s Hospital and all research was performed in accordance with relevant guidelines and regulations. Informed consent was obtained from all participants’ parents and/or their legal guardians.

### Imaging Parameters

A 3 T GE HDX Scanner was used for all scans. We used an 8-channel infant head coil that was installed within the Nomag transport incubator (Lammers Medical Technology, Germany). Single shot echo planar DTI was acquired in 30 non-collinear directions using the following parameters: TE/TR 86/6000 ms, field of view 160 mm, matrix 256 × 256; slice thickness 2.4 mm, and b = 1000 s/mm^2^; time = 5 min, 12 sec. We also acquired an axial T2-weighted fast spin echo: TE/TR = 142/10000 ms; FOV 160 mm, matrix 320 × 256; slice thickness 2 mm; time = 4 min, 30 sec. A pediatric neuroradiologist examined all structural MRI scans using a standardized scoring system^7,41^.

### Structural MRI

Infants with isolated germinal matrix or intraventricular hemorrhage without ventricular dilation, mild diffuse excessive high signal intensity (restricted to anterior caps or posterior crossroads) or with focal (<5 mm) signal abnormalities/cystic changes were coded as having mild abnormalities on their term-equivalent age MRI. Infants with intraventricular hemorrhage with ventricular dilation, moderate ventriculomegaly without hemorrhage, extensive (>5 mm) signal abnormalities/cystic changes, moderate-severe diffuse excessive high signal intensity (affecting several regions including subcortical white matter), or a 2 to 4 weeks’ delay in gyral maturation were defined as having moderate abnormalities. Infants were defined as having severe abnormalities if they exhibited parenchymal hemorrhage/periventricular venous infract, bilateral cystic periventricular leukomalacia, severe hydrocephalus/ventriculomegaly, or a greater than 4-week delay in gyral maturation on their term-equivalent age MRI.

### Data Analysis

#### Image Pre-Processing

**Figure 5:**
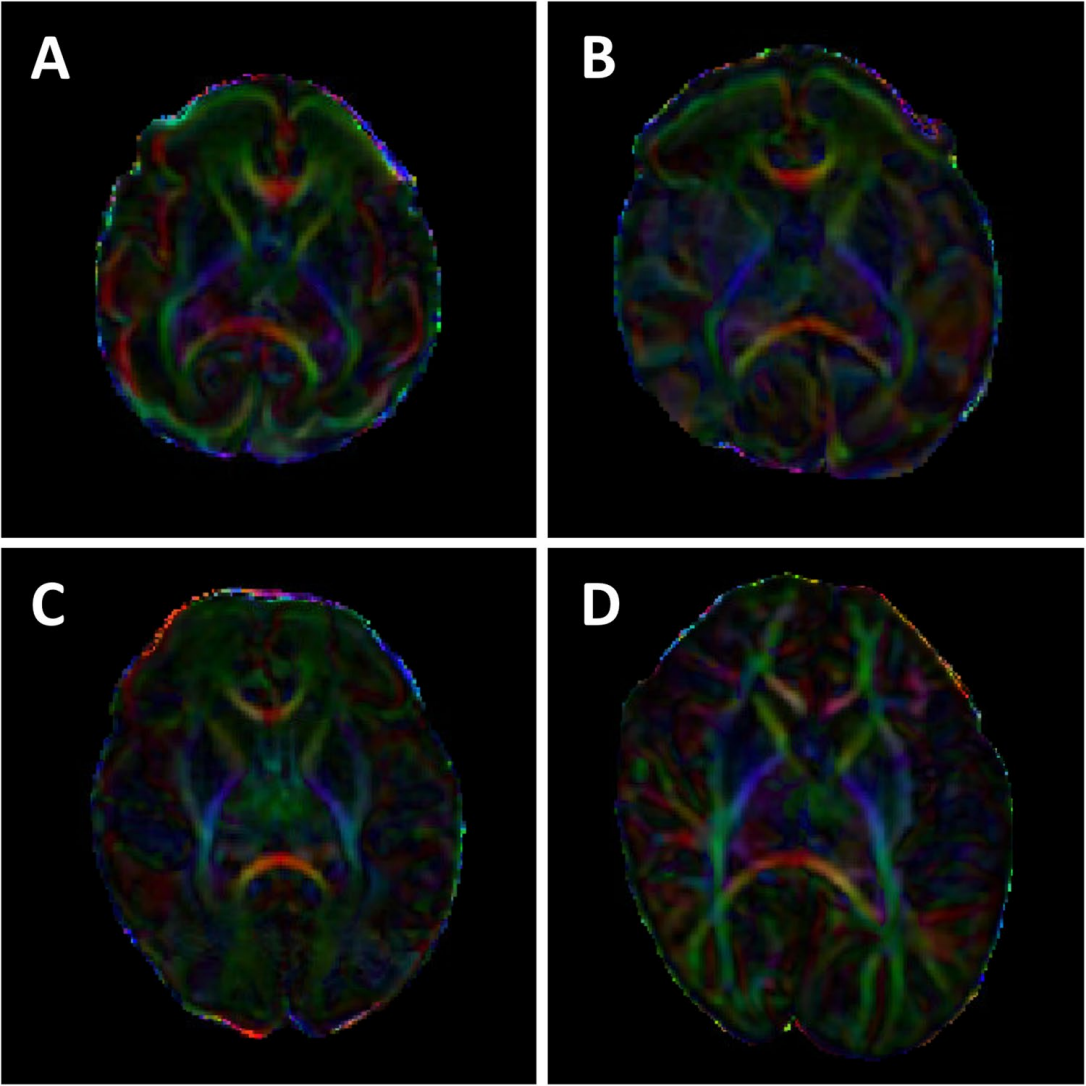
Fractional anisotropy color maps. A representative 28 weeks’ gestational age very preterm infant’s FA color maps at postmenstrual age of (**A**) 29, (**B**) 33, (**C**) 38 and (**D**) 53 weeks.

DTI data were transferred to an offline workstation for further image processing and analysis. We pre-processed all images using FSL 5.0 software of FMRIB Software Library (Analysis Group, FMRIB, Oxford, UK) and DTIStudio 3.0.2 of MRI Studio (Johns Hopkins University, Baltimore, MD). Briefly, we converted raw DTI DICOM data into analyze format using DTI Studio and imported this into FSL to perform eddy current correction. To further minimize the effects of motion and artifacts, we performed Automatic Image Registration (AIR) and automatic outlier slice rejection in DTI Studio. Next, we performed, tensor estimation and generated scalar maps (FA, MD, radial diffusivity [RD], and axial diffusivity [AD; L1 – lamda1]). Figure 5 illustrates examples of FA color maps at each of the four postmenstrual age time points. Last, in FSL, we employed the Brain Extraction Tool to perform brain extraction and BEDPOSTX (Bayesian Estimation of Diffusion Parameters Obtained using Sampling Techniques) to run probabilistic tractography and model for crossing fibers. BEDPOSTX creates all the files for running probabilistic tractography by employing Markov Chain Monte Carlo sampling to build up distributions on diffusion parameters and model crossing fibers within each voxel of the brain^59^. This software permits inclusion of datasets with up to 60 diffusion directions.

#### Image Post-Processing

**Figure 6:**
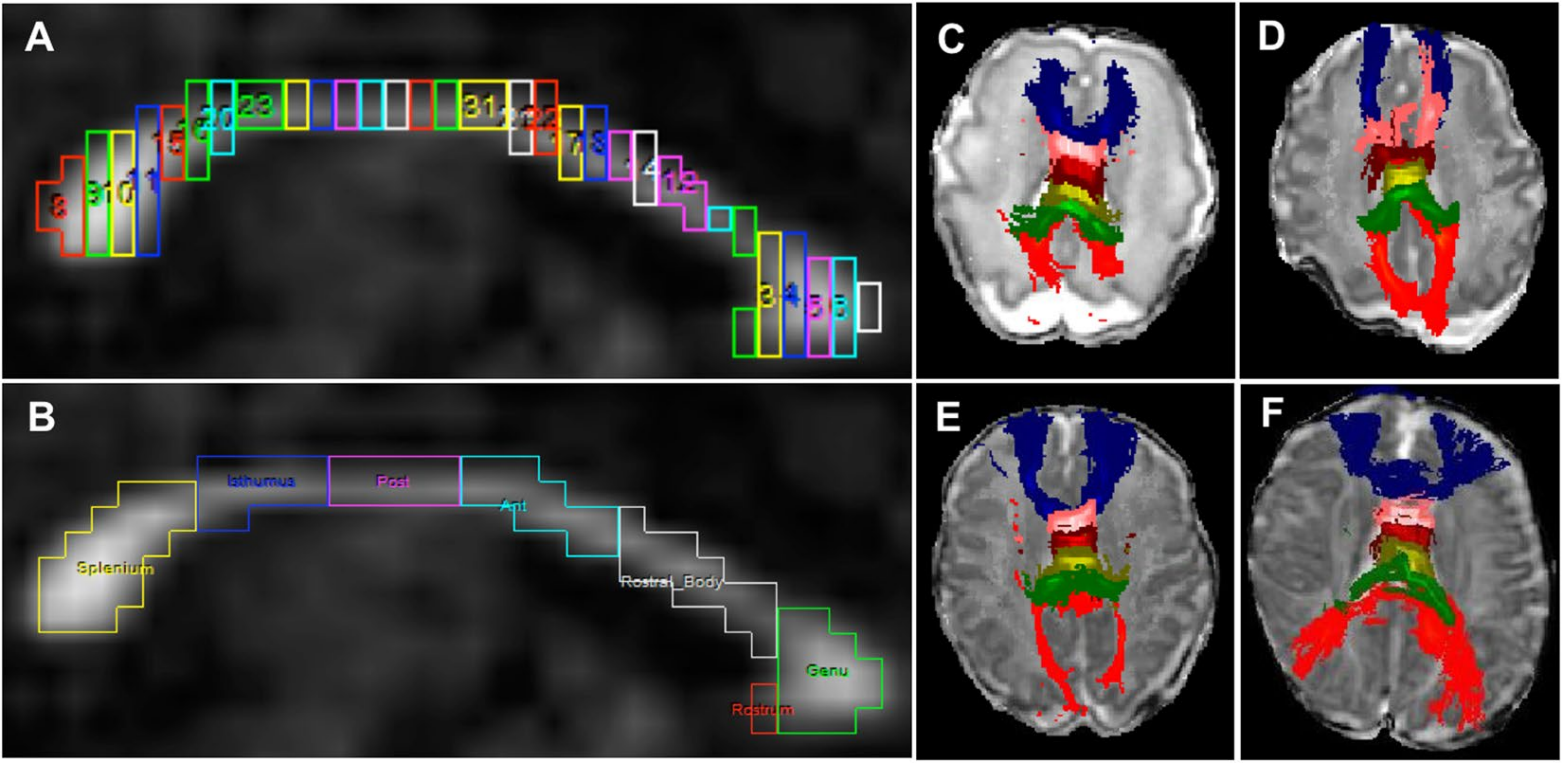
Segmentation and tractography of the corpus callosum (CC) subregions. (**A**) Representative image of CC segmentation on a mid-brain slice on sagittal orientation divided into 30 vertical segments of equal width. (**B**) Relabeling of the 30 segments into seven CC subregions. Because the smallest subregion, the rostrum (red), was not fully developed/visible in all infants, this structure was combined with the genu. (**C**) Probabilistic tractography of the genu (blue), rostral body (pink), anterior midbody (burgundy), posterior midbody (light green), isthmus (green), and splenium (red) overlaid on a diffusion B0 image in axial orientation in a 29-week gestational age preemie imaged at 30 weeks postmenstrual age. (**D,E,F**) Tractography in the same 29 weeks preterm infant imaged at 33, 38, and 53 weeks postmenstrual age, respectively.

**Figure 7:**
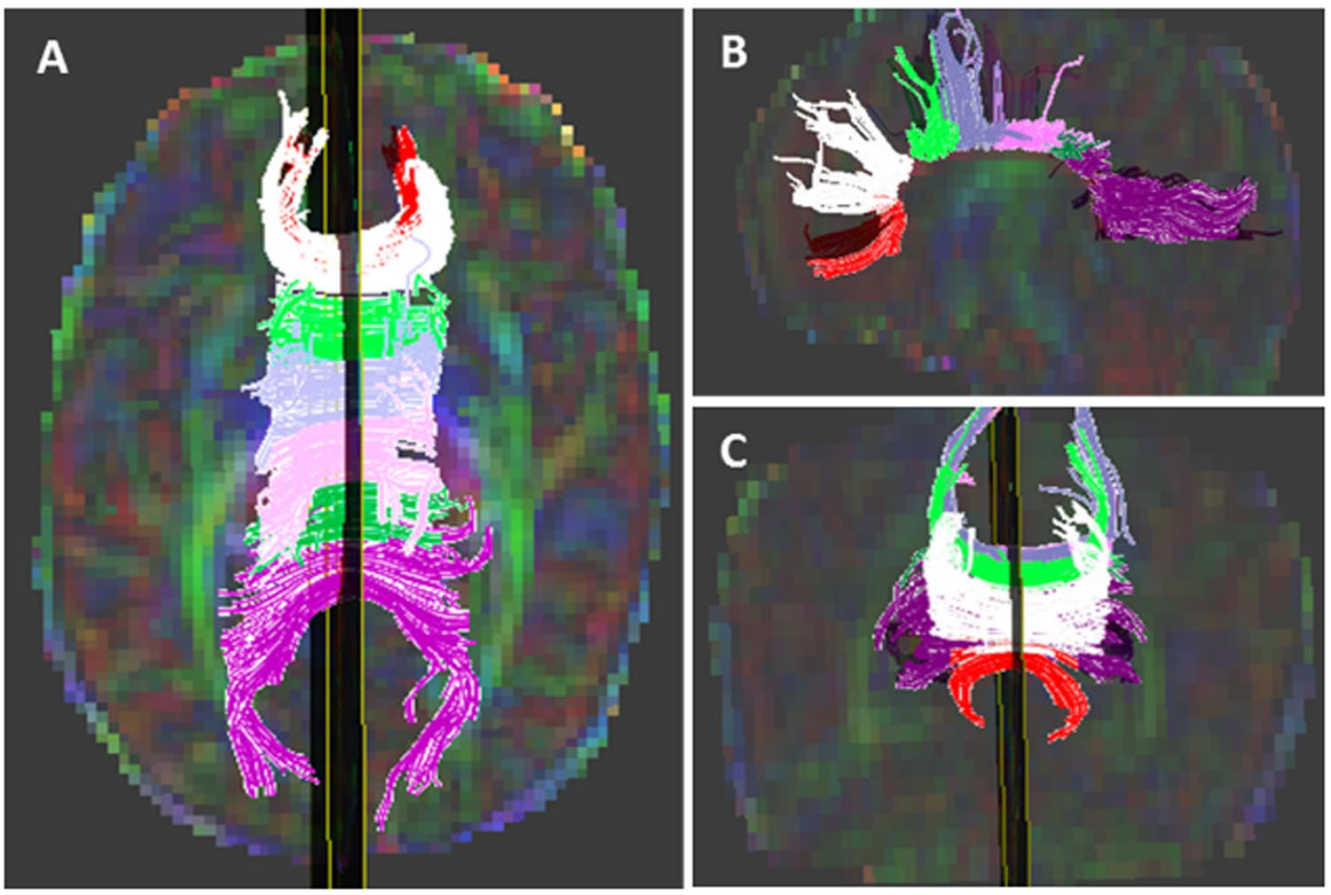
Tractography of the corpus callosum (CC) subregions in three orientations. Tractography of the rostrum (red), genu (white), rostral body (light green), anterior midbody (light blue), posterior midbody (pink), isthmus (green), and splenium (purple) overlaid on a diffusion color map in axial (**A**), sagittal (**B**), and coronal (**C**) orientations in a 29-week gestational age preemie imaged at 38 weeks postmenstrual age.

Using the “ROI Tool” in Analyze 12.0 software (Mayo Clinic), the CC bundle was segmented on the most clearly delineated midbrain slice in the sagittal orientation. The “Auto-Trace” tool was selected to trace out the CC and divide it into 30 vertical segments of the same width (Fig. 6A). This allowed us to divide the CC into six component parts, as described by Thompson *et al*.^25^. Of the 30 segments, proceeding in an anterior to posterior direction, the first 5 segments were labeled as the genu (5/30), the next 5 as the rostral body (RB) (5/30), followed by anterior midbody (AMB) (5/30), posterior midbody (PMB) (5/30), isthmus (4/30), and splenium (6/30) (Fig. 6B). The rostrum (red segment adjacent to genu in Fig. 6B) was typically small or unidentifiable in many very preterm infants and therefore was included as part of the genu whenever it was visible^25^. Last, we imported each of the six newly labeled subregions of the CC as a seed point mask into FSL’s probabilistic tracking with crossing fibers (PROBTRACKX) tool to perform probabilistic tractography (Figs 6C–F and 7). With the exception of using 0.4 mm for the step length (due to the smaller infant brains), we used the default parameters suggested by FSL developers^59^. To ensure good inter-rater reliability, two raters segmented a different set of cases prior to these study cases to establish consistency in methodology. The raters then independently segmented the CC subregions of a random sample of 15 cases to measure inter-rater reliability.

### Clinical Factors

Clinical variables were chosen based on a systematic search in PubMed/Medline of previous literature using Medical Subject Headings, including “Corpus Callosum/growth and development”, “Infant, Premature”, “magnetic resonance imaging”, “risk factors”, “Diffusion Magnetic Resonance Imaging”, and “Neural Pathways/pathology”. Based on this search, we found the following perinatal risk factors were significantly associated with CC and/or NDI: maternal age, maternal education, gestational age/PMA, birth weight, sex, low Apgar score at 5 minutes, total days of breast milk received in first 28 days after birth, BPD, and duration of caffeine therapy^3,4,6,10,13,23–25,27,28,33,36–38,40,54,62^. Each variable was defined as previously described^33,38^ and correlated with our four DTI scalars.

### Statistical Analysis

We measured four DTI parameters (FA, MD, AD, and RD) for the six different regions of the CC. Linear mixed effect models with random intercept and random slope were used to determine the effects of covariates on the trajectory of each parameter, controlling for PMA at MRI scan. PMA at MRI scan was retained in all models, even when insignificant, to control for the effects of age at MRI scan. The fitted mean values and their 95% confidence intervals based on linear mixed effect models were plotted. A quadratic term for PMA at MRI was evaluated in each model to permit fitting for non-linear data, if present; it was removed from the model when insignificant. The values for “Mean FA/RD/AD Difference” in Table 3 are the beta coefficients for all significant (p < 0.05) clinical variables that correlated with CC subregion diffusion metrics. For continuous variables, this value reflects the change in diffusion metric for each unit increase in the clinical variable (e.g. one year increase in maternal age). Data for the AD and RD before 30 weeks PMA lacked sufficient variability thus causing the linear mixed effect model for each of these scalars to not converge. Therefore, we excluded these 20 subjects for the first MRI time point for just the AD and RD trajectory models. Two-sided P-values of less than 0.05 were considered to indicate statistical significance. We corrected for multiple comparisons using the Hochberg procedure to decrease the false discovery rate^63^.

To assess inter-rater reliability, we calculated the inter-class correlation coefficient (ICC) for the PMB and isthmus in the R statistical program using a two-way, consistency-based model. Results were analyzed to determine the consistency and reliability of segmentation methods as performed by two raters on a randomly selected subset of 15 cases. Within-subject standard deviation (SD) and repeatability were also determined as additional more robust measures of reliability. The within-subject SD is defined as the common SD of repeated measurements and calculated by obtaining the square root of the mean within-subject variance^64^. Repeatability is defined as 2.77 times the within-subject SD and is the 95% interval for change between two or more repeat measurements.

### Data availability statement

All of the data generated or analyzed during this study are included in this published article. They are also available from the corresponding author on reasonable request.
